# Multi-method dating reveals 200 ka of Middle Palaeolithic occupation at Maras rock shelter, Rhône Valley, France

**DOI:** 10.1038/s41598-024-69380-w

**Published:** 2024-09-03

**Authors:** Maïlys Richard, Miren del Val, Helen Fewlass, Virginie Sinet-Mathiot, Philippe Lanos, Edwige Pons-Branchu, Simon Puaud, Jean-Jacques Hublin, Marie-Hélène Moncel

**Affiliations:** 1 Archéosciences Bordeaux, UMR 6034 CNRS-Université Bordeaux Montaigne, Pessac, France; 2 Centro Nacional de Investigación Sobre la Evolución Humana, Burgos, Spain; 3 Department of Early Prehistory and Quaternary Ecology, University of Tübingen, Tübingen, Germany; 4 Max Planck Institute for Evolutionary Anthropology (MPI-EVA), Leipzig, Germany; 5 Department of Anthropology and Archaeology, University of Bristol, Bristol, UK; 6 Univ. Bordeaux, CNRS, Ministère de la Culture, PACEA, UMR 5199, Pessac, France; 7 Univ. Bordeaux, CNRS, Bordeaux INP, CBMN, UMR 5248 and Bordeaux Proteome Platform, Bordeaux, France; 8 Laboratoire des Sciences du Climat et de l’Environnement (LSCE/IPSL UMR CEA/CNRS/UVSQ – Université Paris Saclay), Gif-sur-Yvette, France; 9 Centre de Recherche en Archéologie, Archéosciences, Histoire (UMR 6566 CReAAH), Rennes, France; 10 Chaire de Paléoanthropologie, CIRB, Collège de France, Université PSL, CNRS, Paris, France; 11 Histoire Naturelle de l’Homme Préhistorique, UMR 7194 CNRS-Muséum National d’Histoire Naturelle, Paris, France; 12 Géosciences Rennes, UMR 6118 CNRS - Université de Rennes, Rennes, France

**Keywords:** Chronology, Neanderthal, Luminescence, Radiocarbon, Electron spin resonance, Uranium-series, Bayesian modelling, ZooMS, Biogeochemistry, Climate sciences

## Abstract

The emergence of the Middle Palaeolithic, and its variability over time and space are key questions in the field of prehistoric archaeology. Many sites have been documented in the south-eastern margins of the Massif central and the middle Rhône valley, a migration path that connects Northern Europe with the Mediterranean. Well-dated, long stratigraphic sequences are essential to understand Neanderthals dynamics and demise, and potential interactions with *Homo sapiens* in the area, such as the one displayed at the Maras rock shelter (“Abri du Maras”). The site is characterised by exceptional preservation of archaeological remains, including bones dated using radiocarbon (^14^C) and teeth using electron spin resonance combined with uranium series (ESR/U-series). Optically stimulated luminescence was used to date the sedimentary deposits. By combining the new ages with previous ones using Bayesian modelling, we are able to clarify the occupation time over a period spanning 200,000 years. Between ca. 250 and 40 ka, the site has been used as a long-term residence by Neanderthals, specifically during three interglacial periods: first during marine isotopic stage (MIS) 7, between 247 ± 34 and 223 ± 33 ka, and then recurrently during MIS 5 (between 127 ± 17 and 90 ± 9 ka) and MIS 3 (up to 39,280 cal BP).

## Introduction

**Figure 1:**
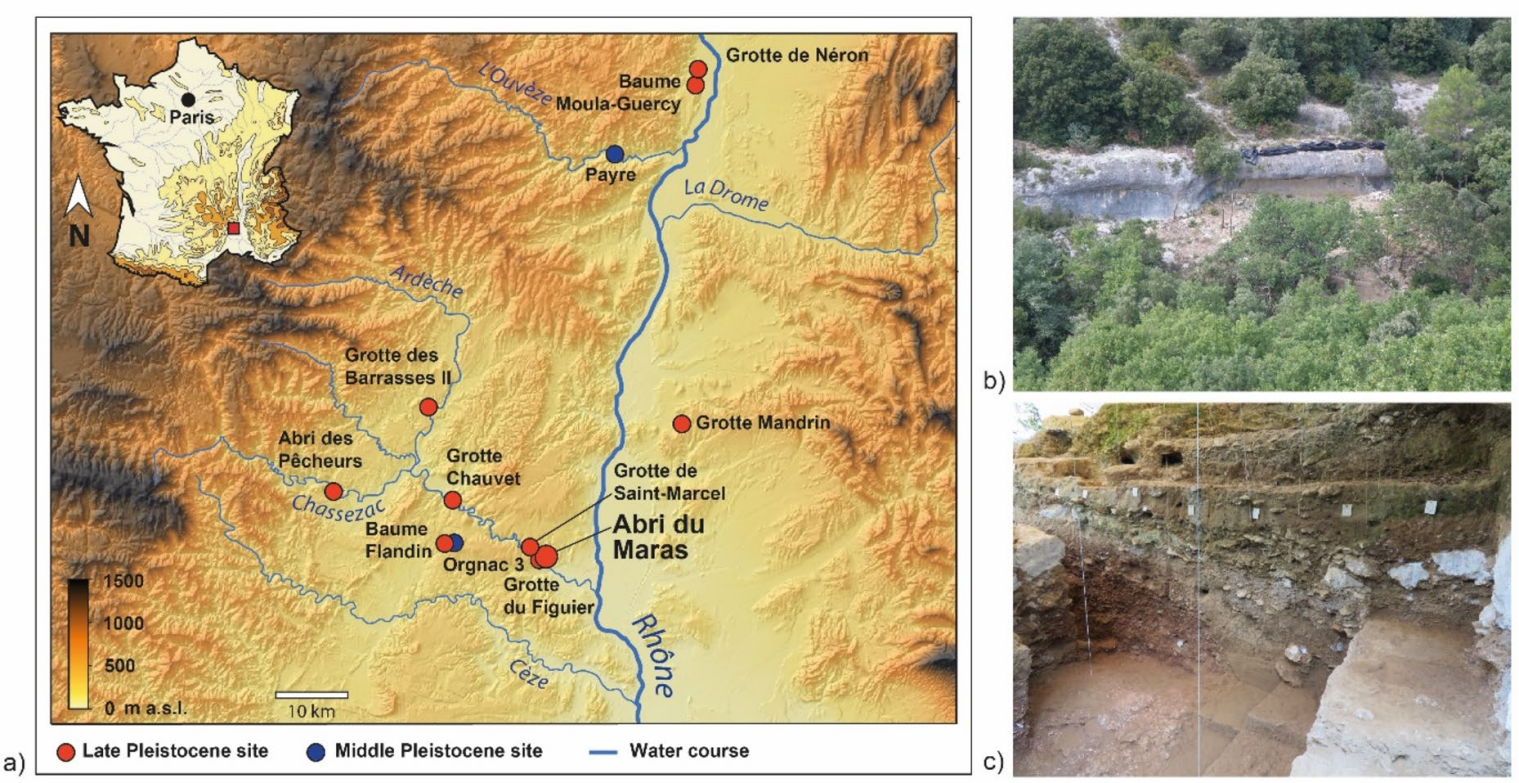
Location of Middle and Late Pleistocene sites in the middle Rhône Valley, France (**a**, modified from^5^), view of the rock shelter (**b**) and of the stratigraphy (**c**).

In south-eastern France, the Rhodanian corridor, which connects the North of Europe and the Mediterranean basin, has been a hotspot for human occupation and used as a migration path starting from the Middle Pleistocene. In particular, in the middle Rhône valley and the south-eastern margins of the Massif central, the earliest evidence of Levallois technology has been documented ca. 300 ka years ago in Orgnac^1,2^ and in Payre^3–5^ (Fig. 1).

The density of sites increased during the Late Pleistocene, where Neanderthal sites are mainly dated to marine isotopic stage (MIS) 5 to 3 (e.g.,^5,6^) and *Homo sapiens* may have been present as early as ca. 54 ka^7^. The earliest evidence of Aurignacian cave art in France is documented at the Chauvet cave, ca. 36 ka (e.g.,^8,9^). In this context, the chronology of the Maras rock shelter is essential for our understanding of population dynamics during this period, considering that Neanderthals and *Homo sapiens* may have crossed paths in this area.

**Figure 2:**
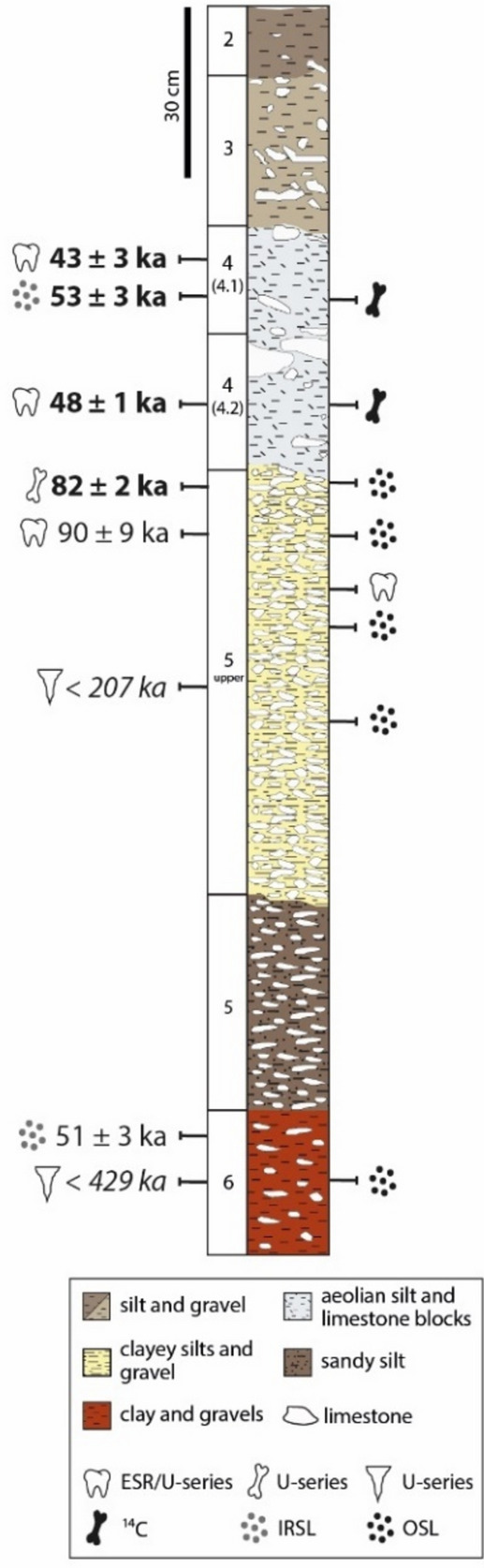
Stratigraphic sequence of Maras rock shelter, with the previous dating results displayed in Table 1 on the left (regular: individual age; bold: weighted mean age; italic: maximum age) and the location of the new samples dated in this study on the right. Note that layer 4 is subdivided into two phases of human occupation (levels 4.1 and 4.2) and that levels 5.1, 5.2 and 5.3 are phases of human occupation of layer 5 upper, present only in the eastern part of the site and thus not represented here.

Thanks to a long-term collaboration in the field between the different specialists working at the site, it has been possible to constrain the chronology as the excavations progressed. Indeed, since the excavations started more than twenty years ago, material for dating has been collected for different methods, including teeth and bones for electron spin resonance (ESR) and uranium-series (^230^Th/U) dating, sediment for infrared stimulated luminescence (IRSL) of feldspar and soda straw for U-series^5,6, 10^. More specifically, the Middle Palaeolithic level 4.1 located at the top of the sequence, is dated to ca. 46–40 ka^6^, contemporary with the beginning of the Upper Palaeolithic in Europe (e.g.,^11–14^). However, the base of the stratigraphic sequence, which shows similarities with the material excavated at Payre, has not been precisely dated. So far, only a maximum age obtained on soda straw, ca. 429 ka, is available for layer 6^5^ (Fig. 2). This study is focused on constraining the chronology of the lower part of the sequence (layers 6 and 5 upper), using optically stimulated luminescence (OSL) on quartz recovered from the sediment, and ESR/U-series dating on tooth enamel. It also aims at increasing the resolution of the timeline for the upper part of the sequence, combining non-destructive near infrared (NIR) spectroscopy for collagen pre-screening with radiocarbon (^14^C) dating to provide an age for the last occupation period by Neanderthals.

Collagen peptide mass fingerprinting, specifically zooarchaeology by mass spectrometry (ZooMS), has been applied to the fragmentary bone specimens dated through radiocarbon. This complementary method contributes to the accuracy and reliability of contextual documentation in chronological analyses. ZooMS is a minimally destructive proteomic method that focuses on morphologically unidentifiable bone fragments, and can provide a taxonomic identification based on protein amino acid sequence variation through the analysis of collagen protein type I.

These new chronological data are essential to discuss the emergence of the Middle Palaeolithic, and more generally the diachronic variability of Neanderthal behaviour prior to the lasting occupation of the region by *Homo sapiens*, which may have led to their disappearance.

## Maras rockshelter

### Site description

Located in the Ardèche gorge (Fig. 1), the Abri du Maras (44°18′43.4’’N 4°33′46.2"E; 170 m asl) is a rock shelter that was first excavated in the 1950s and 1960s by R. Gilles and J. Combier^15^. Since the 2000s, excavations have been conducted by M.-H. Moncel.

The new stratigraphy is composed of several layers, numbered from 6 (bottom) to 1 (top) (Fig. 2). A detailed description of the stratigraphy and a map showing the different excavation areas can be found in the Supplementary Material (Fig. S1). Two phases of roof collapse occurred between layers 5 upper and 4, and between layers 3 and 2. Most of the archaeological material was recovered in layers 6, 5 upper-5 and 4. The top of the sequence contains a low density of artefacts, possibly disturbed by a sheep pen used in the 1960s. Correlations of the newly excavated sequence in the eastern part of the site with the stratigraphy of J. Combier and R. Gilles^15^ at the western limit of the site are difficult due to differences in depths and thickness between the two areas, due to the erosion of sediments in the western area (Fig. S1 in Supplementary Material). R. Gilles and J. Combier completely excavated layer 1 and no section is now visible.

Limestone bedrock was reached at the front of the present-day shelter and shows a series of steps towards the shelter wall. Cryoclastic elements and coarse deposits were identified at the base of the sequence in layer 6 in a red sediment composed of limestone blocks and red silty-sandy deposits. Small soda straw stalactites indicate a roof was present in the past that is now totally collapsed. Remains of this deposit cover the limestone substratum, preserving it from erosion.

Layer 5 upper is thick and subdivided into three main occupation phases (5.1, 5.2, 5.3) with a higher density of material at the bottom. It rests on layer 5 s.s., which is a stonier deposit. The thickness of layer 5 upper-5 varies according to their location on the site. The greatest thickness (around 70–80 cm) is in the eastern part of the site, composed of brown silty and stony lenses with various extensions. These brown stony layers with a gentle slope are truncated in the front of the site, 10 m from the present-day shelter, eroded in part by the development of the small valley and covered by large blocks coming from the major collapse of the roof. Occupations during layers 5 upper and 5 occurred prior to the first phase of roof collapse, as indicated by the type of deposits, when the cave was larger than the present-day rock shelter.

A layer of between 50 cm and more than 1 m of limestone blocks (corresponding to the first collapse phase of the shelter) separates layer 5 upper-5 from overlying layer 4, the latter being subdivided into levels 4.1 and 4.2, which are the two main phases of human occupation. Layer 4 is composed of poorly sorted blocks of roof spall embedded in a silt matrix with a sterile loess deposit between the two phases of occupation^16–20^. These occupations took place beyond the present-day dripline due to the subsequent collapse of the overhang of the shelter. The more recent occupations, documented at the top of the sequence, took place in a shelter configuration, after the second phase of roof collapse between layers 3 and 2. The deposits documenting the final occupations are sparse due to historical disturbance from the animal pen.

### Archaeological assemblages

The layer 6 assemblage shows a low density of archaeological material, with artefacts made predominantly of quartz and quartzite, with some flint. Cores and flakes are on flint. Quartz pebbles and large quartzite cobbles produced small quartz flakes and large quartzite flakes. Few retouched flakes have been recovered up to now. The faunal remains are highly fragmented.

In layer 5 upper-5, the core technologies are more diversified, and more retouched flakes were uncovered, mainly made of flint coming from southern outcrops^18^. Some evidence of semi-Quina tools is observed in the diversified core technology and evidence of an early Rhodanian Quina facies raises questions. The in-situ core technologies are diversified with discoid, orthogonal, multidirectional and Levallois cores, and cores on flake. The retouches on flakes are more invasive and the ratio of retouched flakes is higher than for the upper phase of occupation. Evidence of *Alnus* root processing is documented in layer 5^21^.

In layer 4, Levallois core technology was used to produce flakes, blades and points, mainly from flint, associated to other types of core technologies, such as discoid-type cores made in situ. Flake-tools are rare, including some imported Quina scrapers made on large and thick flakes. The largest products (points, flakes, blades) were imported unretouched in a tool kit from a 30 km perimeter around the site where flint was collected^16^. The site has provided considerable information regarding Neanderthal behaviour and cognitive capacity; fibre technology was evidenced through residue analyses^22^, in particular the identification of 3-ply cordages in level 4.2^23^, dated using ESR/U-series to 55 ± 2 ka to 40 ± 3 ka^6^.

Regarding faunal remains, herbivores dominate the assemblage. Deer (*Cervus* sp.), roe deer (*Capreolus capreolus*) and wild boar (*Sus scrofa*) are present at the base of the sequence, indicating a forest environment. At the top of the sequence, species such as reindeer (*Rangifer tarandus*), bison (*Bison* sp.), and horse (*Equus* cf. *Germanicus*) are the most abundant, and suggest increasingly cold conditions from the bottom to the top of the sequence^24,25^. In layer 5 upper-5, the corpus is diversified with *Sus scrofa, Capreolus capreolus, Capra ibex, Equus hydruntinus, Dama dama* and *Oryctolagus cuniculus*. No carnivore marks were identified and a great number of cut marks were observed, indicating an anthropic origin of the osseus accumulation. Burnt bone, together with charcoal, were also recovered in layers 4 and 5^18^. Intensive exploitation of natural resources is consistent with a residential campsite for long-term occupation events during a whole season, mainly summer, for layer 5 upper-5^25^, while for layer 4, short-term occupations took place mainly in autumn^18,24, 26^.

### Previous dating

Several dating methods have been applied since the 2000s (Table 1). U-series analyses were conducted on bones and teeth, but since the time between the burial and the uranium uptake in the tissues is unknown, these only provide minimum age estimates^27,28^. For this reason, U-series on fossil teeth is generally combined with electron spin resonance (ESR) to model the U-uptake and obtain more precise ages, providing that no U-leaching occurred during burial time^29^. On the other hand, when applied to soda straws found in sediment, U-series gives a maximum age to the archaeological deposit, considering that these thin stalactites break shortly after precipitation, events that precede their incorporation to the sediment (e.g.,^30^). Luminescence dating was also applied to date the time of sediment deposition: when applied to feldspar minerals, infrared stimulated luminescence gives the age of the last exposure of the sediment to sunlight (“bleaching”), i.e., during transportation prior to deposition in the site^31^.

**Table 1:** Synthesis of chronological data obtained at Maras rock shelter. Note that layers l-m from Moncel and Michel^10^ are equivalent to layer 5 upper and that U-series obtained on soda straw represent a *terminus post quem*.

Layer/Level	Sample #	Method / Material	Age (ka)	References
5 upper (l-m)	AM-C5-K4-107	U-series (alpha spectrometry) / bones	87 ± 5	Moncel and Michel^10^
5 upper (l-m)	AM-C5-L1-116		89 ± 4	
5 upper (l-m)	AM-C5-L3-134		91 ± 4	
5 upper (l-m)	AM-C5-M2-155		72 ± 3	
4/4.1	AM L6-221	ESR/U-series / tooth enamel	46 ± 3	Richard et al*.* ^6^
4/4.1	AM L6-229		40 ± 3	
4/4.2	AM L6-769		42 ± 3	
4/4.2	AM G6-203		46 ± 6	
4/4.2	AM J6-393		55 ± 2	
5 upper	AM F6-47		90 ± 9	
4/4.2	AM L11-412	ESR/U-series / tooth enamel	42 ± 3	Richard et al*.* ^5^
4/4.2	AM M10-369		42 ± 6	
4/4.1	WLL922	IRSL / feldspar	46 ± 4	Richard et al*.* ^5^
4/4.1	WLL923		63 ± 3	
6	WLL924		51 ± 3	
5 upper	AM L10-300	U-series (MC-ICPMS) / soda straw*	sup 500	Richard et al*.* ^5^
5 upper	AM M6-1199		sup 500	
5 upper	AM L8-153		207 ± 3	
5 upper	AM L10-307		377 ± 12	
5 upper	AM M8-524		370 ± 14	
5 upper	AM M6-1632		sup 500	
5 upper	AM L10-170		sup 500	
5 upper	AM N6-1001		389 ± 17	
5 upper	AM O9–27		sup 500	
5 upper	AM O9-23		497 ± 48	
5 upper	AM M10-554		434 ± 23	
5 upper	AM L8-249		sup 500	
6	AM U10-503		429 ± 32	
6	AM L13-46		sup 500	
6	AM L14-05		sup 500	
6	AM L13-45		sup 500	
6	AM M12-74		sup 500	

Sup 500 = Secular equilibrium has been reached thus precluding age calculation (samples older than 500 ka).

Previous dating results allowed framing Neanderthal occupation at the site between MIS 5 and 3 (Table 1). In particular, U-series provided a minimum age for bones from layer 5 upper, ranging from ca. 90 to 70 ka^10^. Soda straws from layer 6 and 5 upper gave a maximum age ranging from 429 ± 32 ka (layer 6) to 207 ± 3 ka (layer 5 upper)^5^. ESR/U-series on fossil teeth yielded ages from 90 ± 9 ka (layer 5 upper) to 40 ± 3 ka (layer 4.1). IRSL applied to multi-grain feldspar aliquots provided ages from 51 ± 3 ka (layer 6) to 46 ± 4 ka (level 4.1 in layer 4). These data indicate that human occupation occurred during MIS 5 at the base of the sequence (layer 5 upper) and during MIS 3 at the top of the sequence (levels 4.2 and 4.1), suggesting that MIS 4 is either not recorded at the site or was eroded.

## Results

### Radiocarbon and palaeoproteomics

Thirty-five bone fragments belonging to large herbivores were non-destructively pre-screened with NIR spectroscopy to assess their collagen preservation prior to destructive sampling, and 11 were selected for collagen extraction. The pretreatment information and radiocarbon ages obtained are presented in Table 2. The collagen yields from the pretreated bones were low (0.8–3.9%), as indicated by the NIR pre-screening, but fall within the range expected for material of this age, with only one below the ~ 1% minimum requirement for dating^32^. The elemental values of all extracts fall within accepted ranges of well-preserved collagen (C: ~ 30–50%; N: 11–15%; C:N: 2.9–3.6)^33–35^ with no indications of contamination.

**Table 2:** Pretreatment and radiocarbon data from bones from Maras.

AM #	Level	Material	% Collagen	δ^13^C (‰)	δ^15^N (‰)	%C	%N	C:N	ETH lab num	C14 BP	Error	1σ cal BP (68.3%)		2σ cal BP (95.4%)	
												From	To	From	To
I6-223	4.1	Reindeer tibia	1.1	− 20.0	6.0	42.5	15.1	3.3							
L6-289	4.1	Reindeer tibia	1.0	− 19.4	7.0	35.7	12.6	3.3	ETH-118369	35,880	280	41,220	40,740	41,480	40,450
M6-318	4.1	Reindeer tibia	1.5	− 20.2	5.6	37.4	13.2	3.3	ETH-118370	35,420	260	40,920	40,290	41,090	39,930
M6-421	4.1	Reindeer tibia	1.5	–	–	–	–	–							
E7-208	4.2	Reindeer tibia	2.2	− 19.6	3.9	40.1	14.2	3.3							
H6-241	4.2	Bison tibia	3.9	− 19.7	4.8	42.5	15.2	3.3	ETH-118371	41,070	510	44,530	43,410	44,840	43,080
L6-1001	4.2	Bison tibia	1.2	− 20.0	5.1	36.8	12.9	3.3							
M6-1085	4.2	Bison tibia	1.0	− 19.6	6.6	40.5	14.2	3.3	ETH-118372	34,560	240	39,970	39,430	40,360	39,280
M7-166	4.2	Cervid tibia	0.8	–	–	–	–	–							
F6-24	4.2	Reindeer femur	1.9	− 19.6	5.2	42.2	14.7	3.3	ETH-118373	36,440	300	41,720	41,200	41,950	41,000
M10-542	4.2	Bison	3.6	− 19.5	5.9	40.5	14.4	3.3	ETH-111907	37,990	240	42,390	42,170	42,500	42,040

Note that 35 anthropogenically modified bones were screened for collagen preservation prior to analysis; 11 were selected for collagen extraction and 10 also analysed through ZooMS to determine the taxon (Table 3). Six were chosen for dating.

Two bone collagen extracts from level 4.1 and four from level 4.2 were selected for dating with accelerator mass spectrometry (AMS). The six ^14^C dates fall in MIS 3, between 44,840 and 39,280 cal BP (at 95.4% probability) from level 4.2 and 41,480–39,930 cal BP from level 4.1 (Table 2). The dates obtained are in good agreement with the previous ESR/U-series ages on tooth enamel from the same levels (Table 1). The ^14^C dates were obtained from anthropogenically modified material, indicating human occupation during this interval, and provide a more precise chronological range for the layer 4 assemblage than the dates previously obtained from ESR/U-series.

Four dates from level 4.2 (ETH-111907, ETH-118371, ETH-118372 and ETH-118373) have a spread of ages that do not overlap at the 95.4% range, whereas the two dates from level 4.1 (ETH-118369 and ETH-118370) are statistically indistinguishable (X^2^ test: T = 1.238 (5% 3.841), df = 1). However, the age of M6-1085 (ETH-118372) in level 4.2 overlaps with the dates from 4.1, indicating it may have moved into 4.2 from the sublayer above. However, there are no other indications of movement (anatomical bones were found in connection, lithic refits are present in the same level, ashes lenses were documented undisturbed).

As the bone fragments had been morphologically identified as belonging to large herbivores, they were analysed with ZooMS to determine the genus/species. ZooMS spectra obtained from ten of the bone specimens selected for collagen extraction were taxonomically identified as *Rangifer tarandus* (reindeer, NISP = 4), Cervid/*Saiga* (NISP = 3) and *Bos*/*Bison* (NISP = 3) (Table 3). Collagen type I peptide marker series can be similar for some closely related species, which is notably the case for the species belonging to the following taxonomic groups: *Bos*/*Bison* and Cervid/*Saiga*.

**Table 3:** ZooMS taxonomic identification of the bone specimens analysed for radiocarbon dating.

ZooMS sample #	Square	Specimen ID	Level	R-EVA number	Sample weight (mg)	ZooMS Barcode ID
AM-281	I6	223	4.1	3736	15	*Rangifer tarandus*
AM-282	L6	289	4.1	3737	17.8	*Rangifer tarandus*
AM-283	M6	318	4.1	3738	19.1	*Rangifer tarandus*
AM-284	M6	421	4.1	3739	12	*Rangifer tarandus*
AM-285	E7	208	4.2	3740	11.4	Cervid/*Saiga*
AM-286	H6	241	4.2	3741	14	*Bos*/*Bison*
AM-287	L6	1001	4.2	3742	22.6	*Bos*/*Bison*
AM-288	M6	1085	4.2	3743	14.1	Cervid/*Saiga*
AM-289	M7	166	4.2	3744	13.1	*Bos*/*Bison*
AM-290	F6	24	4.2	3746	12.5	Cervid/*Saiga*

Cervid/*Saiga* can be attributed to either *Cervus elaphus* (red deer), *Megaloceros giganteus* (giant deer), *Alces alces* (elk), *Dama* sp. (fallow deer) or *Saiga tatarica* (saiga antelope). Taxonomic identifications provided by ZooMS align with those obtained through comparative morphology for six specimens. However, four specimens initially classified as reindeer (*n* = 2), cervid (*n* = 1), or Bison (*n* = 1) have been re-identified as Cervid/Saiga (*n* = 3) and Bos/Bison (*n* = 1). This underscores the value of applying ZooMS alongside morphological methods for a more comprehensive assessment of the context of these dated specimens.

### Trapped-charge dating

**Figure 3:**
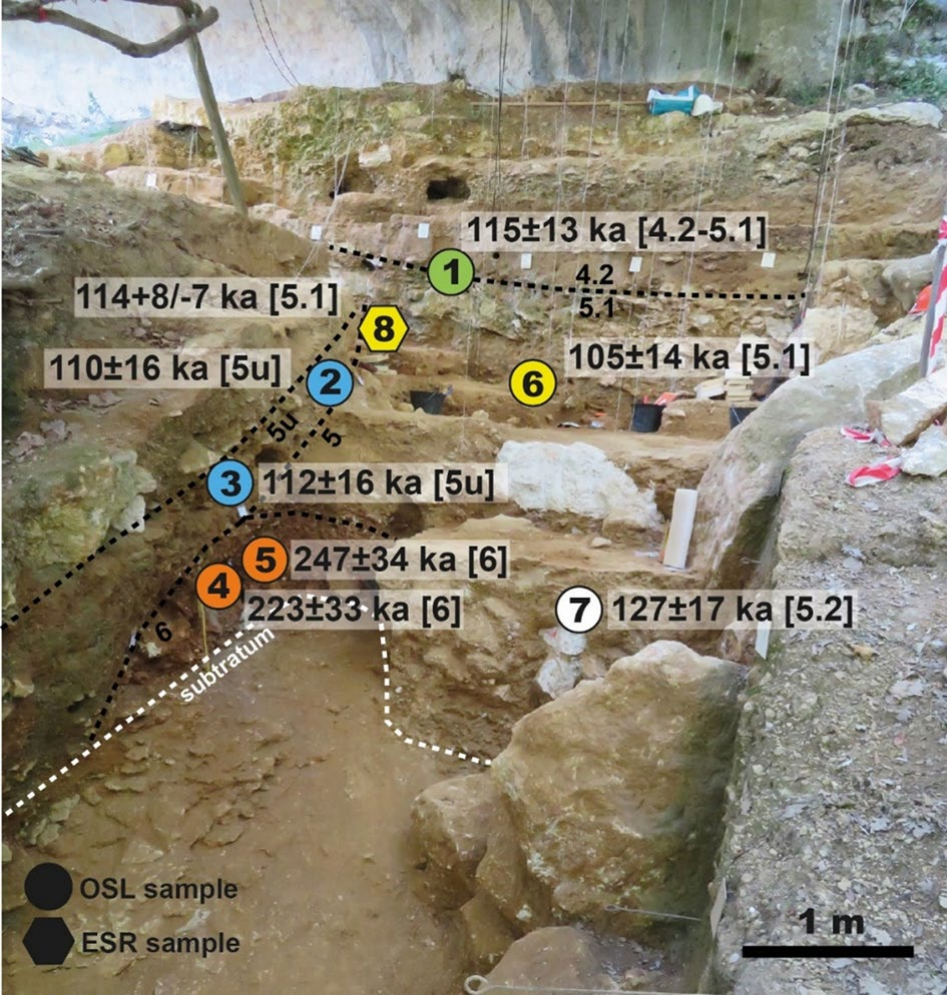
Location and ages (1σ) of the OSL samples (circles) and of the ESR/U-series sample (hexagon) in layers 6 (orange), and 5 upper (blue), and levels 5.2 (white), 5.1 (yellow) and the transition between 5.1 and 4.2 (green).

**Figure 4:**
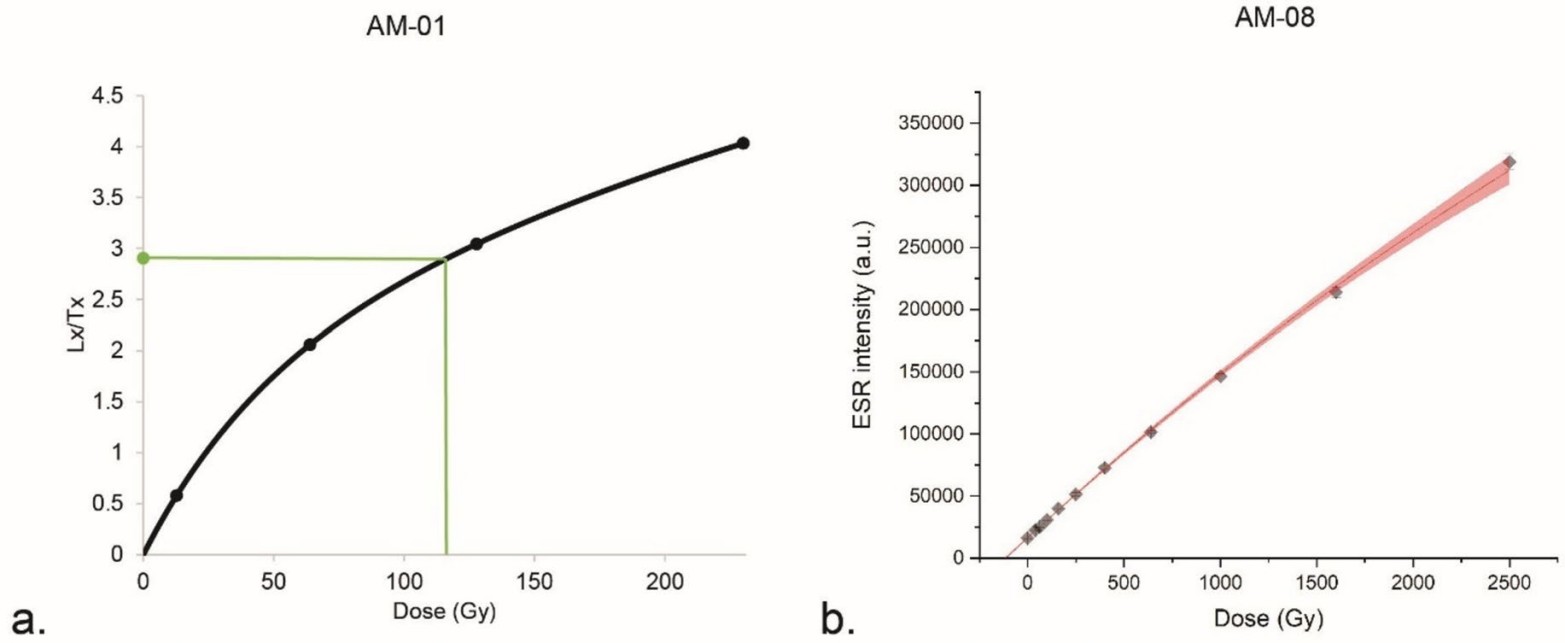
Dose response curves obtained on quartz (**a**: AM-01) and on hydroxyapatite (**b**: AM-08), of 124 ± 6 Gy and 115 ± 3 Gy respectively.

OSL ages are presented in Table 4 and Fig. 3 and were obtained following the measurement protocol in Table 8: they range from 247 ± 34 ka for layer 6 at the base to 115 ± 13 ka for the transition between levels 5.1 and 4.2. According to the preheat (PH) plateau test results, a temperature of 220 °C was selected for preheating the samples, for which the best recovery ratio of 0.88 ± 0.05 was obtained on AM-06. The dose recovery test (DRT) conducted on AM-01 and AM-06 (nine aliquots each) gave ratio values of 0.85 ± 0.09 and 0.90 ± 0.08 respectively. The equivalent doses obtained are included between 211 ± 8 and 127 ± 4 Gy, with overdispersion (OD) values ranging from 25 to 11% respectively (Table 4). An example of dose response curve is given in Fig. 4. In general, samples are homogeneous, only three samples out of seven have OD values ≥ 21%. For this reason, the central age model (CAM^36^) was used for age calculation. Regarding the environmental dose rate, similar values were obtained within a layer; however, across the sequence, dose rate values range from 767 ± 64 (layer 6) to 2018 ± 97 µGy∙a^-1^ (layer 5.1). The heterogeneity of the dosimetric environment is due to the nature of the dated layers and particularly to the presence of numerous blocks of limestone that affect the dose rate (Fig. 3).

**Table 4:** OSL results (1 σ) presented as a function of the stratigraphic order.

Sample #	Layer/ Level	Square	*n*	D_e_ (Gy)	OD (%)	Dose rate (µGy∙a^-1^)				Age (ka)
						Beta	Gamma	Cosmic	Total	
AM-01	4.2–5.1	K5/K6	21	127 ± 4	11 ± 2	581 ± 16	454 ± 7	70 ± 10	1105 ± 20	115 ± 13
AM-02	5u	K8/L8	18	162 ± 7	16 ± 3	920 ± 27	493 ± 71	63 ± 10	1476 ± 77	110 ± 16
AM-03	5u	K9/L9	18	160 ± 8	18 ± 4	876 ± 21	491 ± 71	57 ± 10	1424 ± 75	112 ± 16
AM-06	5.1	L5/L6	45	211 ± 8	25 ± 3	1146 ± 33	809 ± 90	63 ± 10	2018 ± 97	105 ± 13
AM-07	5.2	N9/N10	46	180 ± 6	21 ± 2	849 ± 21	518 ± 72	52 ± 10	1419 ± 76	127 ± 17
AM-04	6	K9/L9	18	171 ± 12	27 ± 5	392 ± 10	323 ± 61	52 ± 10	767 ± 64	223 ± 33
AM-05	6	K9/L9	22	199 ± 7	15 ± 3	440 ± 10	314 ± 60	52 ± 10	806 ± 62	247 ± 34

The equivalent doses (D_e_) were calculated using the central age model (CAM^36^). 5u = 5 upper; *n* = number of accepted aliquots, OD = overdispersion.

Considering the 1 σ error, the OSL ages follow the stratigraphic order and are in agreement within each layer. A weighted mean age can be calculated for layers 6 and 5 upper, for which two samples were analysed. They are 235 ± 33 ka and 111 ± 16 ka respectively. Level 5.2 is dated to 127 ± 17 ka and 5.1 to 105 ± 13 ka. An age of 115 ± 13 ka was obtained for the transition between levels 5 and 4.2. In general, ages from layer upper 5 (obtained on the western part of the site) and level 5.2 and 5.1 on the eastern part (layer 5 upper), and the transition to level 4.2 (eastern part of the site, layer 4) fall in the same range. Indeed, the resolution of the OSL ages (ca. 10%) does not allow differentiating phases of human occupation within MIS 5 for this part of the sequence.

The combined ESR/U-series age (1 σ) is given in Table 5 and shown in Fig. 3. The equivalent dose is 115 ± 3 Gy, and the dose response curve is shown in Fig. 4. Due to low U-content in the enamel (Table 6), the main contribution to the dose rate comes from the sediment. Isotopic ratios ^234^U/^238^U and ^230^Th/^234^U gave apparent U-series ages of ca. 28 ka (enamel) and ca. 47 ka (dentine), describing a recent (*p* = 1.84 ± 0.23) and close to linear uptake (*p* = 0.22 ± 0.12) respectively. Moreover, the contamination in exogeneous Th, assessed through the measurement of ^230^Th/^232^Th, is negligible, with ratios > 147^37^. An ESR/U-series age of 114 + 8/-7 ka was obtained for AM-08, in agreement with the OSL age of 105 ± 13 ka obtained for level 5.1 (AM-06), confirming a MIS 5 chronology for this part of the sequence.

**Table 5:** ESR/U-series dating results (1 σ) for tooth sample AM-08.

Sample #	Level	Square	D_e_ (Gy)	Dose rate (µGy/a)					Age (ka)
				Enamel (α + β)	Dentine (β)	Sediment (γ + cosmic)	Sediment (β)	Total	
AM-08	5.1	K7	115 ± 3	36 ± 7	115 ± 21	774 ± 47	87 ± 12	1011 ± 53	114 + 8/−7

U-uptake was reconstructed using the ESR-US model^29^, which takes into account U-content, ^234^U/^238^U and ^230^Th/^234^U in the enamel and the dentine (see Table 6).

**Table 6:** U-series results used for U-uptake modelling and corresponding *p*-values.

Tissue	Labcode	[^238^U] ppm	[^232^Th] ppb	(^234^U/^238^U)	(^230^Th/^234^U)	(^230^Th/^232^Th)	Apparent age (ka)	*p*-value
Enamel	9761	0.73 ± 0.01	3.59 ± 0.03	1.0725 ± 0.0011	0.2230 ± 0.0007	147.3 ± 0.5	27.5 ± 0.1	1.84 ± 0.23
Dentine	9821	33.21 ± 0.27	4.20 ± 0.03	1.0575 ± 0.0011	0.3507 ± 0.0008	8890.4 ± 18.7	47.0 ± 0.2	0.22 ± 0.12

Apparent ages are not used in the age calculation but are provided for information, as minimum age estimates for each dental tissue.

### Bayesian modelling

**Figure 5:**
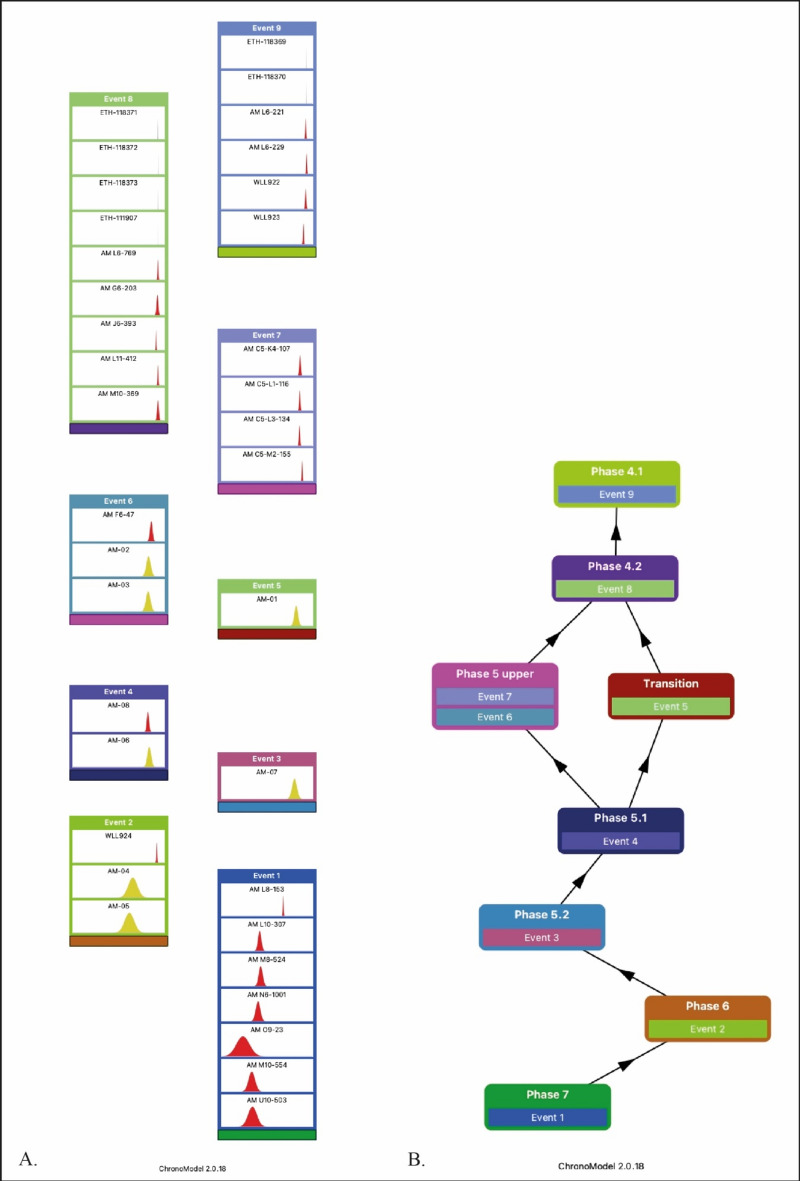
(**A**) Events model: the 9 Events each contain between 1 and 9 dates which are assumed to be contemporaneous within each Event; (**B**) Phases model: the 8 Phases contain 1–2 Events. Stratigraphic relationships are represented by arrows going from the oldest to the most recent phase.

ChronoModel version 2.0.18 was used to model 36 dates from layers 6 to 4 (level 4.1) (Tables 1, 2, 4 and 5, and Supplementary information). This set of ^14^C, ESR/U-series, OSL, IRSL and U-series dates are grouped into 9 Events (Fig. 5a), which are divided into 8 phases (Fig. 5b).

**Figure 6:**
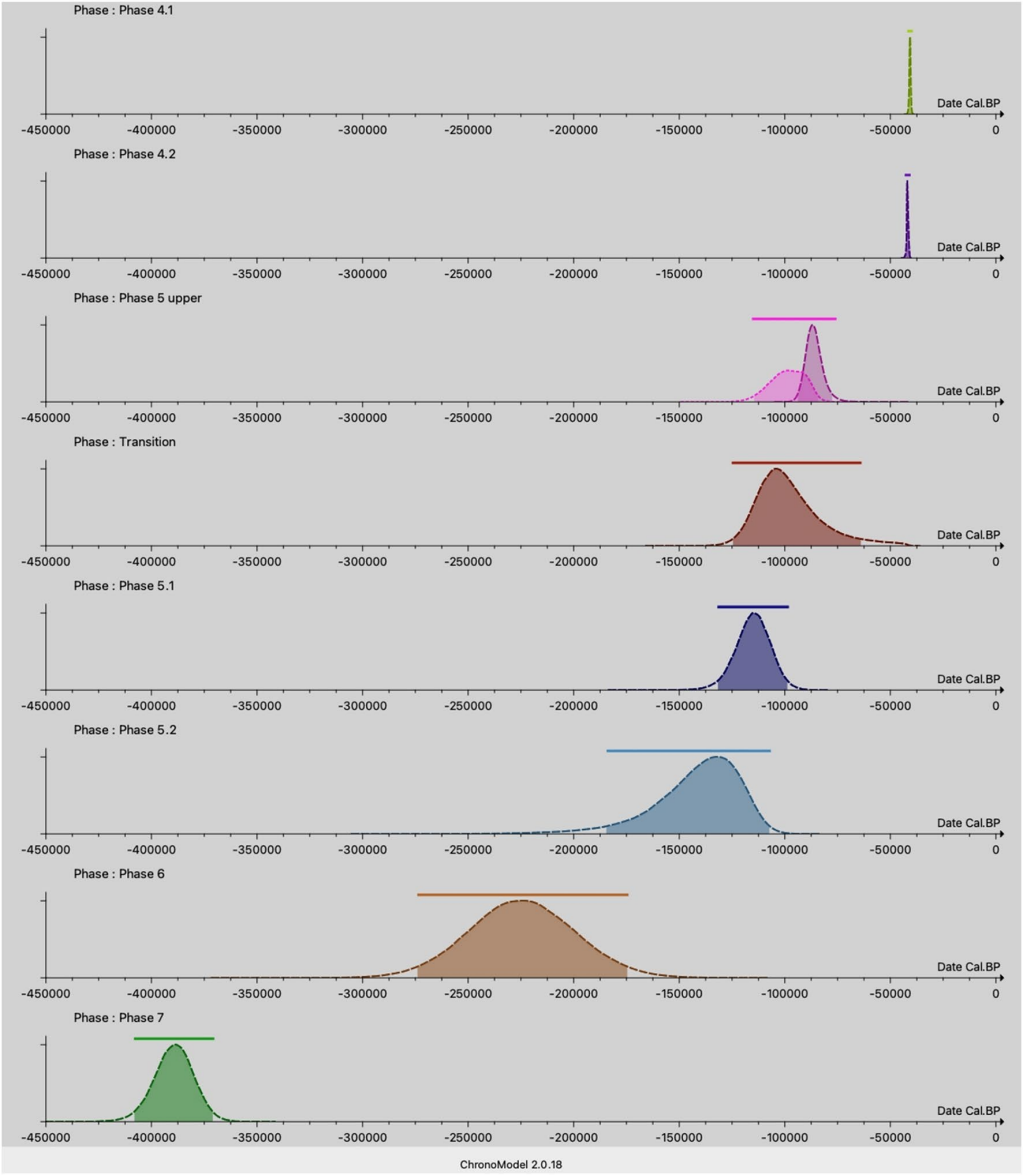
Chronological phasing of the Maras rock shelter: a posteriori date distribution of the beginning and end of each of the 8 phases characterised. The time scale used is cal. BP (before 1950). For phases with only one Event, the start and end distributions are identical. Only phase “5 upper” has two different start and end distributions because it contains two events.

The Event model is based on the assumption that all the dates it contains are contemporary with the event we aim to date (target date). Each event cannot represent a prolonged duration of time. Differences between dates are explained by experimental errors and calibration errors, as in the case of radiocarbon dating. However, larger discrepancies can occur, the origin of which is usually unknown, making a date appear to be an outlier. The property of the Event model is that it can penalise this outlier without the need to make any additional assumptions, and without the need to eliminate it from the analysis. On the other hand, the temporal constraints imposed by the stratigraphy (arrows on the phase graph in Fig. 5b) also penalise outliers if they are in a stratigraphic inversion position. In the present model, there is only one event per phase, except for the "5 upper" phase, which contains two events. Consequently, the estimates for the beginning and end of each phase will be identical, except for the "5 upper" phase. Figure 6 shows the distribution over time of the a posteriori “begin” and “end” of the eight phases of the model, after the Bayesian model has been calculated.

The numerical results are presented in Table 7 and are expressed in calibrated BP (before 1950). This table provides the chronology of the Maras shelter for the phases concerned. The APM column corresponds to the a posteriori mode: this is the date that corresponds to the maximum of the a posteriori date distribution. The HPD begin—HDP end columns give the "Highest Posterior Density" date interval obtained at the 95% confidence level. The APM dates fix the chronology on the time scale, while the HPD intervals indicate the uncertainties surrounding them at the 95% confidence level.

**Table 7:** Summary table of a posteriori statistical results obtained after Bayesian chronological modelling with ChronoModel 3.

Item	Phase	APM	HPD begin	HPD end
Event 9	Phase 4.1	− 40,879	− 41,616	− 40,154
Event 8	Phase 4.2	− 42,191	− 42,790	− 41,167
Event 7	Phase 5 upper	− 87,317	− 94,250	− 79,147
Event 6	Phase 5 upper	− 98,635	− 116,300	− 79,736
Event 5	Phase Transition	− 104,162	− 124,553	− 64,557
Event 4	Phase 5.1	− 114,367	− 131,739	− 99,092
Event 3	Phase 5.2	− 132,415	− 184,192	− 107,337
Event 2	Phase 6	− 226,011	− 273,989	− 174,589
Event 1	Phase 7	− 388,227	− 408,098	− 371,116
Phase 5 upper	Start	− 98,388	− 114,626	− 84,276
Phase 5 upper	End	− 87,200	− 94,010	− 77,689

Dates are expressed in cal BP. APM column: a posteriori mode determined on the a posteriori date distribution. HPD begin—HDP end columns: "Highest Posterior Density" date interval obtained at the 95% confidence level. Note that since Phase 5 upper contains two events (6 and 7), the beginning and the end of the phase can be estimated using Chronomodel. The other phases contain only one event, the beginning and the end of each phase corresponds to the event itself.

In the supplementary material, Figs. S2, S3, S4 and S5 give details of the results a posteriori, Event by Event. This makes it easy to identify the outliers and to see how the date distribution of the Event is positioned in relation to the dates that make it up: distributions with a grey line for the individual calibrated dates and distributions calibrated by the global model (in colour). When there is only one date in the Event, there may still be an offset: this is due to the action of the stratigraphic constraints of the global model.

## Discussion

**Figure 7:**
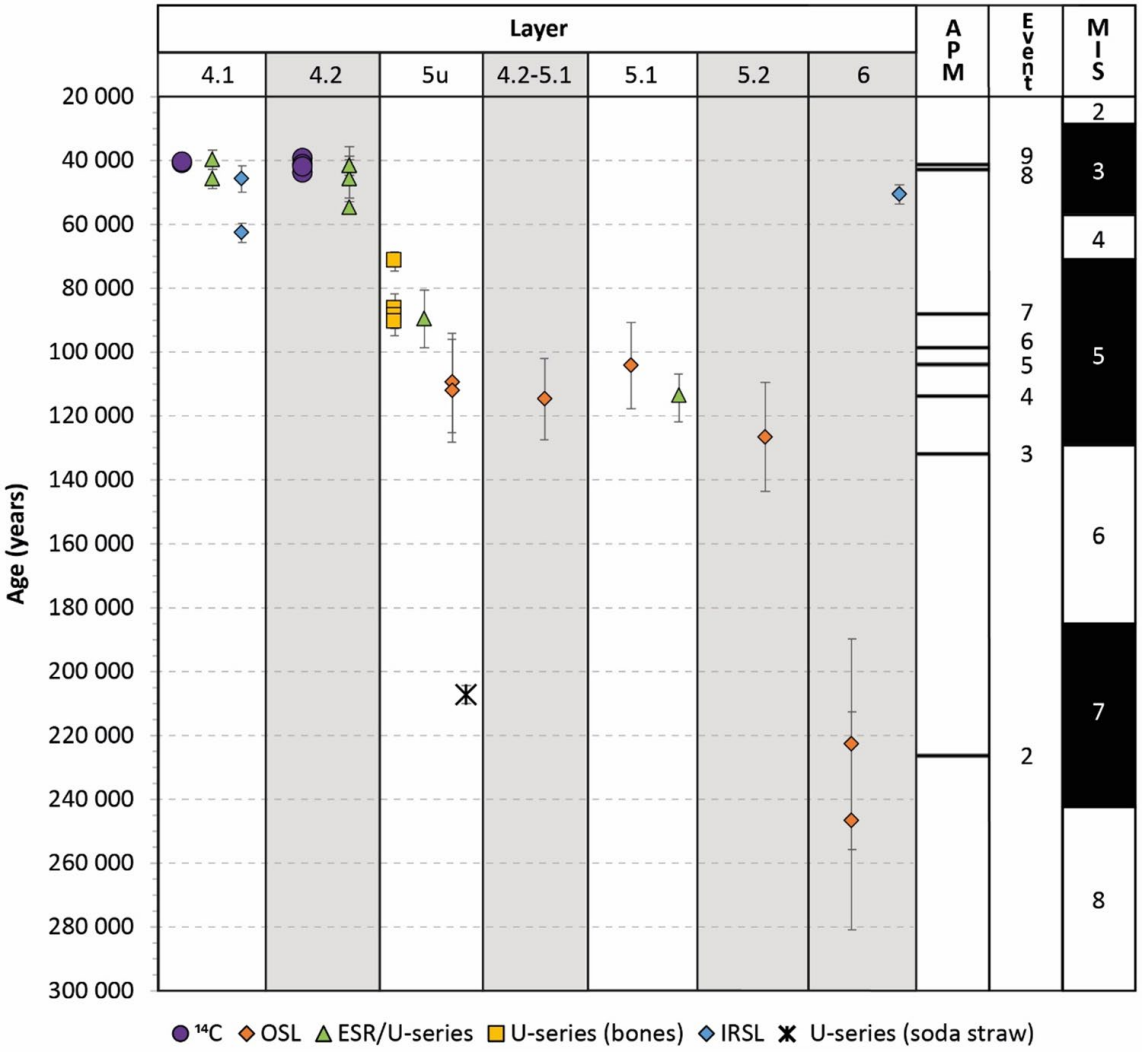
Synthesis of the dating results (1σ) obtained at Abri du Maras. APM (a posteriori mode) for each event are represented, as well as corresponding MIS (marine isotopic stage). Note that due to the scale, the error bars of the radiocarbon dates are not visible, and only the youngest U-series date obtained for layer 5 upper can be represented and thus the APM (388 227 years) as well as the corresponding event (1) are not visible on the figure.

Since 2000, a total of 36 ages have been obtained at the Maras rock shelter, following a multi-method and multi-material approach (see Tables 1, 2, 5, 6 and Fig. 7). In general, the age results are in good agreement, considering that the data obtained on the soda straws represent a maximum estimate. Two out of three IRSL age results do not follow the stratigraphic order, 63 ± 3 ka (WLL 923, layer 4.1) and 51 ± 3 ka (WLL 924, layer 6). At the time of the sampling, no in-situ dosimetry could be conducted, and considering the high heterogeneity of the deposits due to the presence of limestone blocks, the calculated environmental dose rate, based on a sediment sample (ca. 100 g), may be inaccurate (see discussion in^5^).

The new ESR/U-series age of 114 + 8/ − 7 ka obtained on level 5.1 (top layer 5 upper) is in agreement with OSL ages of 115 ± 13 ka (AM-01, at the transition between levels 4.2 and 5.1) and 105 ± 13 ka (AM-06, level 5.1) that indicate an MIS 5 chronology for this layer. These ages are slightly older than those obtained using U-series on bones (Fig. 7), but these are generally considered as minimum age estimates (since the time span between the burial and the uranium uptake is unknown, see^38^ and discussion in^5^).

The first OSL ages obtained at the site allow extending the chronology of the site to ca. 250 ka (layer 6 dated from 247 ± 34 to 223 ± 33 ka), suggesting that the base of the sequence at Maras is coeval with human occupation in Payre^4,5^ located north of the area along the Rhône Valley and attributed to the early Middle Palaeolithic. For layer 5 upper, two ages of 110 ± 16 ka (AM-02) and 112 ± 16 ka (AM-03) were obtained for the western part of the site, in agreement with the ESR/U-series age of 90 ± 9 ka obtained on a tooth from the same layer^6^ (Table 1); the ages of 105 ± 13 ka (AM-06, level 5.1) and of 127 ± 17 ka (AM-07, level 5.2) obtained for the eastern part of the site suggest an MIS 5 chronology. Due to the resolution of the ages obtained using trapped-charge dating methods, we are not able to chronologically distinguish these three phases. However, considering all available data for the site, there is no evidence of MIS 4 deposits, which may be due to erosion processes. A similar chronology is obtained for the nearby sites of the Saint-Marcel cave (level u^39^) and the Baume Flandin (level 3^5^), dated to MIS 5e.

The radiocarbon ages obtained for the top of the sequence confirm that Maras rock shelter was occupied by the latest Neanderthals populations, up to ca. 40 ka BP. Late Neanderthal occupations have also been recorded at the nearby cave of Saint-Marcel (ca. 38 ka BP^40^). These ages coincide with the beginning of the Upper Palaeolithic in Europe attributed to *Homo sapiens*, with directly dated *Homo sapiens* recently shown to be present in Germany as early as 47.5 ka BP^14^, suggesting that both groups could potentially have overlapped in this area.

According to the Bayesian model (Fig. 6 and Table 7), and considering the Highest Posterior Density (HPD) date intervals obtained at 95% confidence level, Event 1 (phase 7), dated from the soda straws found in layers 5 upper and 6^5^, corresponds to an early phase when the roof was still present (cave configuration) between ca. 408 and 371 ka, probably before human visited the cave. Event 2 (layer 6), records the earliest human presence at the site, between ca. 274 (MIS 8) and 175 ka (MIS 6). We need to consider that the Ardèche River and the landscape were completely different at the time of the earliest human occupation; more specifically, during these two phases, the site was possibly a big cave covering the whole end of a small valley, dark and humid (hence the presence of the stalagmites and the red sediments). Blocks were found in the red deposits of layer 6, suggesting that the collapse of the roof started when humans first visited the site. Event 3 (level 5.2) indicates that humans likely occupied the rock shelter between 184 ka (MIS 6) and 107 ka (MIS 5) and Event 4 suggests that level 5.1 would be coeval with MIS 5 (ca. 132–99 ka). A hiatus is observed between the levels 5.1–4.2 transition (Event 5, ca. 125–65 ka, documented in the eastern section of the site, see Fig. 3) and layer 5 upper (Events 6 and 7, ca.116–79 ka, documented in the western section), and levels 4.2 (Event 8, HPD 42.8–41.2 ka) and 4.1 (Event 9, HPD 41.6–40.2 ka), which fall within MIS 3. The APM, which represents the maximum of the a posteriori date distribution, is 226 ka for layer 6, 132 ka for level 5.2, 114 ka for level 5.1, 104 ka for the transition between levels 5.1 and 4.2, 99 ka for layer 5 upper, 42.2 ka for level 4.2 and 40.9 ka for level 4.1. All these dates fall within interglacial periods, with the exception of the level 5.2, at the transition between MIS 6 and 5. It is thus possible that sediment from glacial periods may not have been preserved or deposited in the site, either due to its location and configuration, or to the climatic conditions.

In conclusion, our new chronological data obtained at Abri du Maras suggest that Neanderthals were present mainly during three interglacial periods: MIS 7, 5 and 3, as indicated by the fauna. During the MIS 3, they hunted mainly *Rangifer tarandus* with rare secondary species (Bovids and Cervids)^41^. During the MIS 5, human groups hunted a diversified game including both *Rangifer tarandus* and forested species (*Sus scrofa, Dama dama*)^42^ and during the MIS 7, *Cervus elaphus* is the main hunted species (unpublished data). This detailed chronology provides new insights into the timing of the evolution of Neanderthal behaviour at the Maras rock shelter, used as a long-term residence by Neanderthals. Considering that the earliest human presence at the site is dated to ca. 226 ka (APM for layer 6), the human populations from Maras and those from Payre would have been contemporaneous. The site was repeatedly occupied during MIS 5 (layer 5 upper) and 3 (potentially until 39,280 years cal BP). In particular, the ^14^C ages allow us to precisely date the last occupation period by Neanderthals between ~ 41.5 and 39.9 ka, suggesting that these populations would have been among the very last Neanderthal groups settled in the Middle Rhône valley.

## Methods

Based on the previous dating results obtained (Table 1), in particular for the upper part of the sequence that falls within the range of radiocarbon dating, bones from levels 4.1 and 4.2 were selected for ^14^C dating. During the 2021 excavation campaign, one tooth from level 5.1 was collected for ESR/U-series dating, and sediment from layer 5 upper (western part of the site), levels 4.2–5.1 (transition), levels 5.1 and 5.2 (eastern part of the site) and layer 6 were sampled for OSL of quartz grains (Fig. 2).

### Radiocarbon dating

#### Sample selection and pre-screening

Pre-screening and collagen extraction was carried out in the Department of Human Evolution at the Max Planck Institute for Evolutionary Anthropology (Leipzig, Germany). A selection of 35 long bones (tibia and femur) from large herbivores were pre-screened for collagen preservation to identify suitably preserved material for ^14^C dating. Bones were selected from different squares from layer 4 (level 4.1 and 4.2), all bearing signs of anthropogenic modifications. Non-destructive collagen pre-screening was carried out using a LabSpec 4 hi-res near-infrared (NIR) spectrometer (Malvern Panalytical, Germany), following protocols outlined by^43^. NIR screening indicated low levels of collagen preservation (< 5%), as expected for Pleistocene material. Eleven bones were selected for collagen extraction based on their predicted levels (> 3%).

#### Collagen extraction

The surface of the sampled bone was first cleaned with a shot-blaster and between 380 and 590 mg bone was sampled using a dentistry drill with circular drill bit. Collagen was extracted using the protocol described in^44^. Samples were first demineralised in HCl 0.5 M at 4 °C until soft and CO_2_ effervescence stopped, with HCl changed once per week. The demineralised samples were treated with NaOH 0.1 M to remove humic acid contaminants and re-acidified in HCl 0.5 M. The samples were gelatinised in HCl pH3 at 75 °C for 20 h. The gelatin extracts were filtered to remove > 80 µm particles (Ezee filters, Elkay Labs, UK) and ultrafiltered to concentrate the > 30 kDa fraction (Sartorius VivaSpin Turbo 15). Filters were pre-cleaned following^45^. After freeze-drying for 48 h, samples were weighed to determine the % collagen yield (as a proportion of dry bone weight) with a minimum ~ 1% yield requirement.

To assess the quality of each collagen extract prior to dating, ~ 0.5 mg collagen was weighed into a tin cup using a microbalance and analysed on a ThermoFinnigan Flash elemental analyser coupled to a Thermo Delta plus XP isotope ratio mass spectrometer (EA-IRMS). Stable carbon isotope ratios were expressed relative to VPDB (Vienna PeeDee Belemnite), and stable nitrogen isotope ratios were measured relative to AIR (atmospheric N2), using the delta notation (δ) in parts per thousand (‰). Analysis of internal and international standards indicates an analytical error of better than 0.2‰ (1σ). All extracts had elemental values (C%, N%, C:N) falling within the range of modern extracts^32,34, 35^, indicating their suitability for dating.

#### *AMS dating and *^*14*^*C modelling*

Six collagen extracts were selected for dating. The extracts were graphitised^46^ and dated at the Laboratory for Ion Beam Physics at ETH Zurich, Switzerland using a MICADAS accelerator mass spectrometer (AMS)^47,48^. Aliquots of a background bone (> 50,000 BP) were pretreated and dated alongside the samples to monitor lab-based contamination. The AMS measurements of the collagen backgrounds were used in the age correction of the samples, with an additional 1‰ error added as per standard practise. AMS data reduction was performed in BATS^49^. The ^14^C dates were calibrated in OxCal 4.4^50^ using the IntCal20 calibration curve^51^.

### ZooMS

Ten bone specimens from layer 4 (levels 4.1 and 4.2) selected for collagen extraction for radiocarbon dating were also analysed through Zooarchaeology by mass spectrometry (ZooMS) and sampled destructively (between 11.4 and 22.6 mg) to determine the taxon. Samples analysed through ZooMS followed extraction protocols detailed elsewhere^52–54^. Each bone sample was demineralised in 250 µl 0.6 M hydrochloric acid (HCl) at 4 °C for 20 h. Samples were then centrifuged for 1 min at 10 k rpm and the supernatant was removed. The demineralized collagen was rinsed three times in 200 µl of ammonium bicarbonate (50 mM, NH_4_HCO_3_, hereafter AmBic) to be neutralized to pH 8, and 100 µl of AmBic solution was added to each sample. Next, samples were incubated at 65 °C for 1 h. Then, 50 µl of the resulting supernatant was digested with 1 µl of trypsin (0.5 µg/µl, Promega) at 37 °C overnight, acidified using 1 µl of trifluoroacetic acid (20% TFA), and cleaned on C18 ZipTips (Thermo Scientific). Digested peptides were spotted in triplicate on a MALDI Bruker plate with the addition of α-cyano-4-hydroxycinnamic acid (CHCA, Sigma) matrix. MALDI-TOF-MS analysis was conducted at the University of Cambridge, using an UltraFlextreme MALDI-TOF (Bruker) in reflector mode, with matrix suppression up to 500 Da and collected in the mass-to-charge range 700–3500 m/z.

Triplicates of each sample were merged in R v.4.0.5^55^ and MALDIquant v. 1.21^56^. The intensity was smoothed using a moving average and the baseline was removed using the TopHat approach. Then, the replicate spectra were aligned for each sample using SuperSmoother and a signal to noise ratio of 3, and the three replicates were summed to obtain a single spectrum, in addition to another removal of the baseline again using TopHat. Spectra were then exported as .msd files. Taxonomic identifications were made using mMass^57^ through manual peptide marker mass identification in comparison to a database of peptide marker series for all European Pleistocene medium- to large-sized mammals^52^. To assess any potential contamination by non-endogenous peptides, we performed laboratory blanks alongside the samples. These remained empty of collagenous peptides, excluding the possibility of modern laboratory or storage contamination.

### Optically stimulated luminescence (OSL)

OSL was applied on sediment collected at night from the fresh sections (*n* = 7), and kept in light-proof bags (Fig. 3). A 30-cm deep hole was dug at the location of the samples to conduct in-situ gamma dosimetry, using a portable gamma-ray spectrometer multichannel analyser connected to a NaI(Tl) detector (Inspector 1000, Canberra). The gamma dose rate was obtained for each sample following the “threshold” technique^58^. The excavated sediment was kept to derive the beta dose rate from U, Th and K content measured using mass spectrometry. A water content (water/dry mass of sediment %) of 15 ± 5% was used for calculation based on the previous dating conducted at the site^5,6^ and used for age calculation. The equivalent doses were obtained following the measurement protocol in Table 8. The data were processed using Analyst v.4.57^59^. The signal was integrated using the first 0.5 s and background was subtracted from the last 2.5 s. The following criteria were applied for D_e_ selection: a recycling ratio limit of 1 ± 0.1; recuperation < 5% of the natural signal; maximum test dose error of 10%; and a test dose signal > 3 sigma above background. Infrared (IR) depletion assessments were conducted as well^60^. Equivalent doses were obtained using a sum of two exponentials fitting function. OSL ages were calculated with the 1 σ error range, taking into account the published alpha attenuation^61^ and beta absorption^62^ factors, and the updated dose-rate conversion factors^63^. The cosmic dose rate was calculated from the equations of Prescott and Hutton^64^. U-series equilibrium was assumed for dose rate calculation.

**Table 8:** OSL SAR measurement protocol applied to quartz.

Step	Treatment
1	Given dose
2	Preheat (220 °C for 10 s)
3 (Lx)	Blue stimulation for 40 s at 125 °C
4	Given test dose
5	Preheat (200 °C for 10 s)
6 (Tx)	Blue stimulation for 40 s at 125 °C
7	Optical draining (blue stimulation for 40 s at 280 °C)
8	Return to 1

The samples were sieved and the 150–250 µm fraction was chemically treated to eliminate organic matter (using H_2_O_2_) and carbonates (using HCl). Density separation (of 2.62 and 2.70) was done to extract the quartz fraction used for analysis. Single aliquot regeneration (SAR^65^) analyses were conducted on multigrain 2 mm aliquots following the protocol displayed in Table 7. A preheat (PH) plateau test was performed on aliquots of AM06, previously bleached in a solar simulator (Hönle UVACube 400 solar simulator) for 2 h. Measurements were conducted with PH temperature between 200 and 260 °C with 20 °C steps, and a given dose of ca. 120 Gy. Dose recovery tests were conducted on bleached aliquots AM-01 and AM-06, with a given dose of ca. 75 Gy.

### Combined electron spin resonance/uranium-series dating (ESR/U-series)

The tooth AM-08 (square K7, #598) and the embedded sediment were collected during the excavation campaign in 2021. The dental tissues were mechanically separated and cleaned using a dentist drill in order to conduct ESR analysis on the enamel and U-series analyses on bulk enamel and dentine, following the protocol described in^6^. The enamel powder (100–200 µm) was split into eleven aliquots, from which ten were irradiated at increasing doses (40, 64, 100, 160, 250, 400, 640, 1000, 1600 and 2500 Gy) using a ^137^Cs gamma source (Gammacell, CENIEH, Burgos, Spain), and one kept intact to measure the natural ESR intensity. The ESR intensity of the carbonate hydroxyapatite T1-B2 signal^66^ of each aliquot was measured three times using an X-band spectrometer (Bruker EMXmicro-6/1) at the CENIEH, with the following parameters: 10–50 scans, 1 mW microwave power, 1024 points resolution, 10 mT sweep width, 100 kHz modulation frequency, 0.1 mT modulation amplitude, 20.48 ms conversion time, and 5.12 ms time constant. The mean value and associated standard deviation were plotted as a function of the irradiation dose. The dose response curve was constructed using Origin Pro 8 (Origin Lab Corporation, Northampton, USA). The equivalent dose was obtained by extrapolation using a single saturation exponential (SSE) function^67^ and weighted by the inverse of the squared ESR intensity (1/I^2^).

U-series analyses (U and Th content and isotopic ratios ^234^U/^238^U, ^230^Th/^234^U and ^230^Th/^232^Th) were conducted to model the U-uptake using the ESR-US model, for which an uptake parameter is calculated, the p-value^29^. Ranging from -1 (describing an early uptake, i.e., U incorporated soon after burial) to positive values (describing a recent uptake), this model allows deriving the dose rate individually for each dental tissue. The chemical analyses were performed at the Laboratory for Science of Climate and Environment (LSCE, Gif-sur-Yvette, France) following the procedure for separation and purification of U and Th isotopes of^6^. U and Th fractions were combined for the measurement on a Multi-Collector inductively coupled plasma mass spectrometer (MC-ICP-MS) Thermo Scientific TM Neptune Plus fitted with a jet pump interface and a desolvating introduction system (aridus II) following^68^. ^238^U, ^235^U, ^236^U and ^229^Th were measured on Faraday cups, ^234^U and ^230^Th on an ion counter (see details in^69^).

U, Th and K content from the sediment containing the tooth was determined using ICP-MS to derive the beta and gamma dose rate. A value of 15 ± 5% was used for calculation based on the previous ESR dating conducted at the site^5,6^. For the dental tissues, a water content (weight %) of 0% for the enamel and of 7 ± 5% for the dentine was assumed. The cosmic dose rate was calculated considering sediment and limestone cover above each sample following Prescott and Hutton^64^. Combined ESR/U-series calculation were performed using the DATA program^70^, which takes into account an alpha efficiency of 0.13 ± 0.02^71^ and Monte-Carlo beta attenuation factors^72^. U-series equilibrium was assumed for dose rate calculation (dental tissues and sediment).

### Bayesian modelling

The ChronoModel Application is intended to provide tools for constructing chronologies in archaeology and geosciences in combining Events, Phases and temporal constraints. Models can be developed including data from any dating methods (^14^C, TL/OSL, AM, typo-chronology, etc.) and from archaeological and environmental contexts (stratigraphy, ordering between phases, duration or hiatus constraints)^73,74^. A user-friendly interface is available for entering the data and MCMC (Markov Chain Monte Carlo) calculations can be carried out and inspected in detail, with models and results presented using the Bayesian statistical framework. ChronoModel is a free and open-source cross-platform software (Mac, Windows, Linux)^75^. It is available for downloading at: https://chronomodel.com/. The source code can be downloaded and compiled; the project is hosted on GitHub.com. The repository can be cloned by typing: https://github.com/Chronomodel/chronomodel.git. In the present study, the MCMC calculation was carried out with 3 chains of 1 million iterations each, which ensures the convergence of the calculations, i.e., a good estimate of the a posteriori date distributions (probability density), given the data available.

## Supplementary Information

Supplementary Information.

## Data Availability

The datasets generated during and/or analysed during the current study are available from the corresponding author on reasonable request.
