# Supplementary material for: Multi-method dating reveals 200 ka of Middle Palaeolithic occupation at Maras rock shelter, Rhône Valley, France
Source: Sci Rep. 2024 Sep 3;14:20474. doi: 10.1038/s41598-024-69380-w (PMC11372155; doi:10.1038/s41598-024-69380-w)
Supplement: Supplementary file 1 — Supplementary Information. [file 41598_2024_69380_MOESM1_ESM.pdf]

Supplementary material

Multi-method dating reveals 200 ka of Middle Palaeolithic occupation at Maras rock shelter, Rhône Valley, France

Maïlys Richard[1,2,3]*, Miren del Val[2], Helen Fewlass[4,5], Virginie Sinet-Mathiot[4,6,7], Philippe Lanos[1,8], Edwige Pons-Branchu[9], Simon Puaud[10], Jean-Jacques Hublin[4,11], Marie-Hélène Moncel[12]

[1]Archéosciences Bordeaux, UMR 6034 CNRS-Université Bordeaux Montaigne, Pessac, France.

[2]Centro Nacional de Investigación sobre la Evolución Humana, Burgos, Spain.

[3]Department of Early Prehistory and Quaternary Ecology, University of Tübingen, Tübingen, Germany

[4]Max Planck Institute for Evolutionary Anthropology (MPI-EVA), Leipzig, Germany.

[5]Department of Anthropology and Archaeology, University of Bristol, Bristol, United Kingdom.

[6]Univ. Bordeaux, CNRS, Ministère de la Culture, PACEA, UMR 5199, Pessac, France

[7]Univ. Bordeaux, CNRS, Bordeaux INP, CBMN, UMR 5248 and Bordeaux Proteome Platform, Bordeaux, France.

[8]Géosciences Rennes, UMR 6118 CNRS - Université de Rennes, Rennes, France

[9]Laboratoire des Sciences du Climat et de l'Environnement (LSCE/IPSL UMR CEA/CNRS/UVSQ – Université Paris Saclay), Gif-sur-Yvette, France.

[10]Centre de Recherche en Archéologie, Archéosciences, Histoire (UMR 6566 CReAAH), Rennes, France.

[11]Chaire de Paléoanthropologie, CIRB, Collège de France, Université PSL, CNRS, Paris, France.

[12]Histoire naturelle de l'Homme préhistorique, UMR 7194 CNRS-Muséum National d'Histoire Naturelle, Paris, France.

*Corresponding author: mailys.richard@u-bordeaux-montaigne.fr

Detailed stratigraphy

The deposits of the rock shelter are 7 metres thick and the lower part rests on top of limestone bedrock. Two main sections provide an overview of the sequence (Fig. S1): (1) a section running northeast-southwest, at the KL boundary (from L, to the NE), and extending from band 8 to band 18 over a length of more than 10 metres; (2) a section covering the upper part of the infill at the boundary of bands 4/5 and 5/6 (from the SE, 6 and 5), from band E to band O, with a total length of 11 meters. The archaeological material is present in layers 6 to 3 of the new excavations (which correspond to the base of the sequence described by J. Combier[14]).

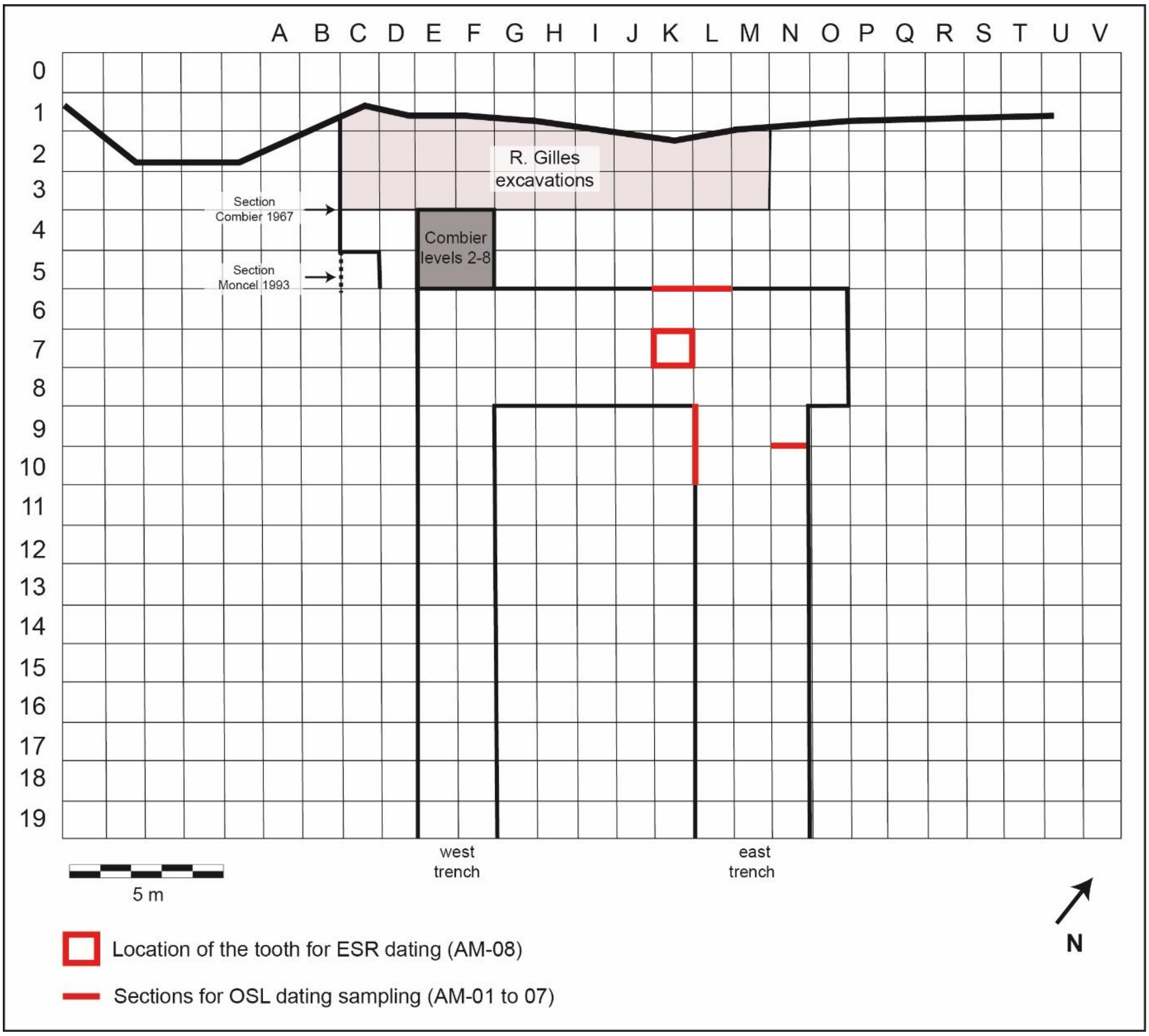

Fig. S1. Site plan showing the area of the 1960s excavations (grey and pink), the new trenches and the location of the OSL (red lines) and ESR (red square) samples displayed in Fig. 2.

- Limestone bedrock

The Urgonian limestone bedrock outcrops at the foot of the section between bands 6 and 18, forming a steep redan from band 11 onwards and then tapering off to form a trough. The lowest point of this trough is between KL13 and 14. The morphology, position and obvious wear of the rock are characteristic markers of past watercourse activity.

- Base complex 1) channelised gravel and red gravel of layer 6

A heterogeneous assemblage of coarse sediments lies on the limestone bedrock, and is visible in the eastern and western trenches. This consists of matrix-poor stones, gravels or coarse sands organised in lenses of variable size, with vertical grading. The base of these lenses is bounded by alignments of larger stones, with subangular edges and smooth surfaces.

Above these coarse sediments, especially between bands K to N, lies a stony facies with a red structured matrix. The thickness of this facies is variable. It is bounded at the top by a line of limestone platelets. This alignment forms an angle of almost 40° towards the valley. This is a semi-open limestone gravel with a reddish-brown silty-clayey matrix. Archaeological material from this horizon was recovered in the eastern trench only.

- Complex 2) Facies of layer 5 (archaeological level) and layer 5 upper (three phases of dense occupation, archaeological levels 5.1, 5.2 and 5.3)

In areas where complex 1 has been eroded, the dark-matrix gravels of Layer 5 lie on the limestone bedrock (Urgonian), which consists of detached platelets. This dark-brown layer becomes increasingly thicker towards the east, reaching a maximum thickness of more than 1 m. The layer is truncated by erosion in band L.

Layer 5 upper rests directly on the bedrock in bands M to O. The facies changes at the top of layer 5 and becomes less stony. It reaches a thickness of almost 30 cm to the west, then expands eastwards to reach a thickness of over 1 m. The coarse limestone fraction consists of elongated platelets with subangular edges and weathered surfaces. The slope is directed towards the valley at an angle of 5° to 15°. The matrix is brown, with a silty-sandy texture (siliceous and carbonated sand). Two lenses of small, compact matrix pebbles are interspersed between bands L to O.

- Complex 3) Silts and collapsed levels

- Sandy silts with granules (layer 4). Archaeological levels 4.1 and 4.2

This layer is visible along the whole length of the section. It is essentially a matrix facies, varying in thickness from 20 to 50 cm. This facies forms the first level of collapsed slabs, representing the first phase of roof collapse. Some lateral facies variations can be observed.

In the south-western part, the facies is essentially silty. In the north-eastern part, the layer 4 facies becomes more heterogeneous. At the top (level 4.1), the matrix facies, with a compact structure and low porosity, is mixed with a coarse fraction of heterometric limestone elements. These elements form alignments defining a coarse trough.

The base and top of the trough are bounded by a silty facies virtually devoid of coarse fraction. The structure of the trough is flat with a gentle slope of a few degrees running towards the bottom of the thalweg.

At the base (level 4.2), the silts remain compact. The few coarse elements scattered in the silts are highly weathered, particularly the granular fraction. Underneath them lies a significant accumulation of rock shelter collapse.

- Silty matrix gravels (layer 3)

Layer 3 is a gravel facies with a silty matrix, with a clear contact and no transition to Layer 4. This continuous brown-coloured layer is between 20 and 30 cm thick.

The coarse fraction is made up of heterometric-sized local limestone elements. These platelets lie flat with a gentle slope towards the bottom of the slope.

- Layer of blocks with a silty matrix (layer 2)

Layer 2 is a heterogenous complex of limestone slabs and heterometric gravels in a silty-sandy matrix. These limestone slabs derive from the second phase of collapse of the shelter’s overhang. They are about a metre long with very subangular edges and traces of dissolution on their surfaces. This layer can be clearly divided into two parts. The lower part presents a pinkish hue and a fairly massive structure. The upper part of the layer, the top of layer 2, has undergone pedogenesis. This horizon, which constitutes the current soil, has taken on a very dark grey-brown hue.

An attempt to reconstruct the sedimentary history of the infill

The shelter lies directly beneath the plateau overlooking the end of the Ardèche Gorges. The present-day valley lies at the foot of the rock shelter, on the left bank of the Ardèche River. The valley slope acts as a trap for sediments over time from the plateau and the slope, as well as for deposits transiting through the valley as they form.

The base of the sedimentary sequence lies on the limestone bedrock and appears to be fractured. A first sedimentary unit (1), in contact with the bedrock, is only visible at the base of

the eastern trench section. The top of this unit appears horizontal, and the transition to the unit above is seamless.

Then, at the base of the two trenches, we observe a sequence of coarse, poorly-sorted levels in a red clayey-silty matrix. The orientation of these elements is clearly marked, with a slope of 20° towards the thalweg. Lateral sedimentary contributions, transiting through the slopes, result from the destabilisation of the latter or from the erosion of possible surface formations on the plateau. The presence of fistulas and the appearance of the sediments indicate the probable presence of a vast cavity or shelter opening on a valley at the same level as the Ardèche River.

The third sedimentary unit (3) consists of layer 5 and layer 5 upper. The facies of these layers vary, but is always a brown gravel matrix. The elements are no longer rolled, but their edges remain subangular. Although these deposits lean against the slope, they tend to be horizontal. Some very imposing boulders are visible in this layer, marking the beginning of the retreat of the overhang of the cave or vast rock shelter.

The first major episode of collapse of the rock shelter's overhang can be seen at the top of this complex. This event is marked in the stratigraphy by an alignment of slabs, often over one metre in size. These slabs correspond to the collapse of the entire stratum that formed the rock shelter's roof.

The last sedimentary unit begins above this remarkable level. It is a homogeneous silty unit but nonetheless contains several fragments derived from the desquamation of the shelter walls. These silty deposits are aeolian loess deposits. They characterise a cold period with sparse vegetation. At the top of Layer 3, a new phase of collapse indicates the further retreat of the shelter's overhang.

In conclusion, the geological study of the sedimentary sequence shows a sequence of deposits with different geomorphological contexts. The site initially formed as a vast cavity or shelter covering the valley bottom (layer 6 with fistulas), then evolved with the collapse of the roof in parallel with the development of the valley and its equilibrium slope connected to the Ardèche River. Layer 6 was largely eroded, and is only preserved in isolated areas on the substratum in the eastern trench. Layers 5 and 5 upper are unconformably deposited and partly eroded at the front of the site, among piles of boulders from the collapse of the rock shelter roof. They are covered by a considerable quantity of collapsed rocks. Layer 4, above this collapse, rests under a rock shelter on the right bank of the valley. It is collapsed in front of the site as a result of the receding roof, and is unconformable with the deposits of layer 5 upper in

front of the site. The sequence is therefore made up of three large, independent, unconformable units, separated by phases of collapse of the cave and then of the rock shelter.

Bayesian chronological modelling with ChronoModel 3

For each of the nine Events, we can make the following observations:

- Event 9 (level 4.1) includes six dates: two ^{14}C (ETH-118369, ETH-118370); two ESR/U-series (AM L6-221, AM L6-229); two IRSL (WLL922, WLL923). Bayesian analysis reveals an outlier: WLL923, which appears to be clearly too old compared with the five other dates (Fig. S1).

- Event 8 (level 4.2) comprises nine dates: four ^{14}C (ETH-118371, ETH-118372, ETH-118373, ETH-111907); five ESR/U-series (AM L6-769, AM G6-203, AM J6-393, AM L11-412, AM M10-369). Bayesian analysis also reveals a main outlier: AM J6-393, which is too old compared to the other eight dates, which are in general consistent with each other, although there is a discrepancy between the two ^{14}C dates (Fig. S2).

- Event 7 (level 5 upper) includes four dates: four U-series (on bones) (AM C5-K4-107, AM C5-L1-116, AM C5-L3-134, AM C5-M2-155). Bayesian analysis reveals an outlier: AM C5-M2-155, a more recent date compared to the other three dates, which are consistent with each other (Fig. S3).

- Event 6 (level 5 upper) includes three dates: one ESR/U-series (AM F6-47); two OSL (AM-02, AM-03). Bayesian analysis shows that the three dates are consistent with each other. No outliers have been identified (Fig. S3).

- Event 5 (transition level 5.1-4.2) includes only one age: one OSL (AM-01). Bayesian analysis shows that this age is slightly younger due to the action of stratigraphic constraints (Fig. S3).

- Event 4 (level 5.1) includes two dates: one ESR/U-series (AM-08); one OSL (AM-06). Bayesian analysis shows that the two dates are consistent with each other, but with a slight ageing of the AM-06 date (Fig. S3).

- Event 3 (level 5.2) includes one age: one OSL (AM-07). Bayesian analysis of this age remains virtually unchanged.

- Event 2 (layer 6) includes three dates: one IRSL (WLL924); two OSL (AM-04, AM-05). Bayesian analysis reveals an outlier: WLL924, an IRSL age that is too young compared with the two other OSL dates, which are also consistent with each other.

- Event 1 (early phase predating layers 5 and 6) includes seven dates: seven U-series on soda straw (AM L8-153, AM L10-307, AM M8-524, AM N6-1001, AM O9-23, AM M10-554, AM U10-503). Bayesian analysis reveals one main outlier: AM L8-153, a date that appears too young compared with the six other dates, three of which are older (shaded curves) and can also be considered as outliers: AM O9-23, AM M10-554 and AM U10-503.

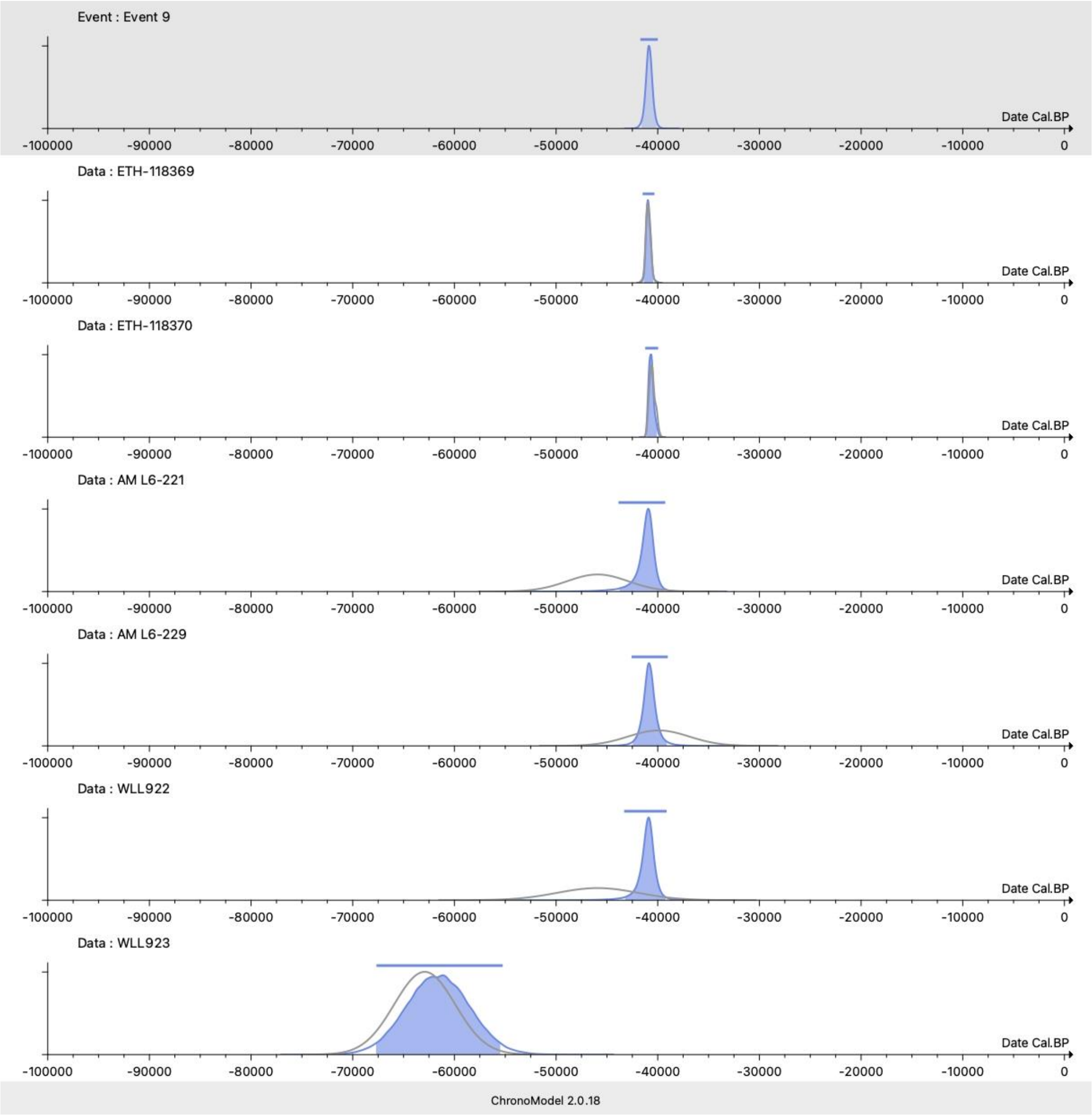

Fig. S2. Event 9.

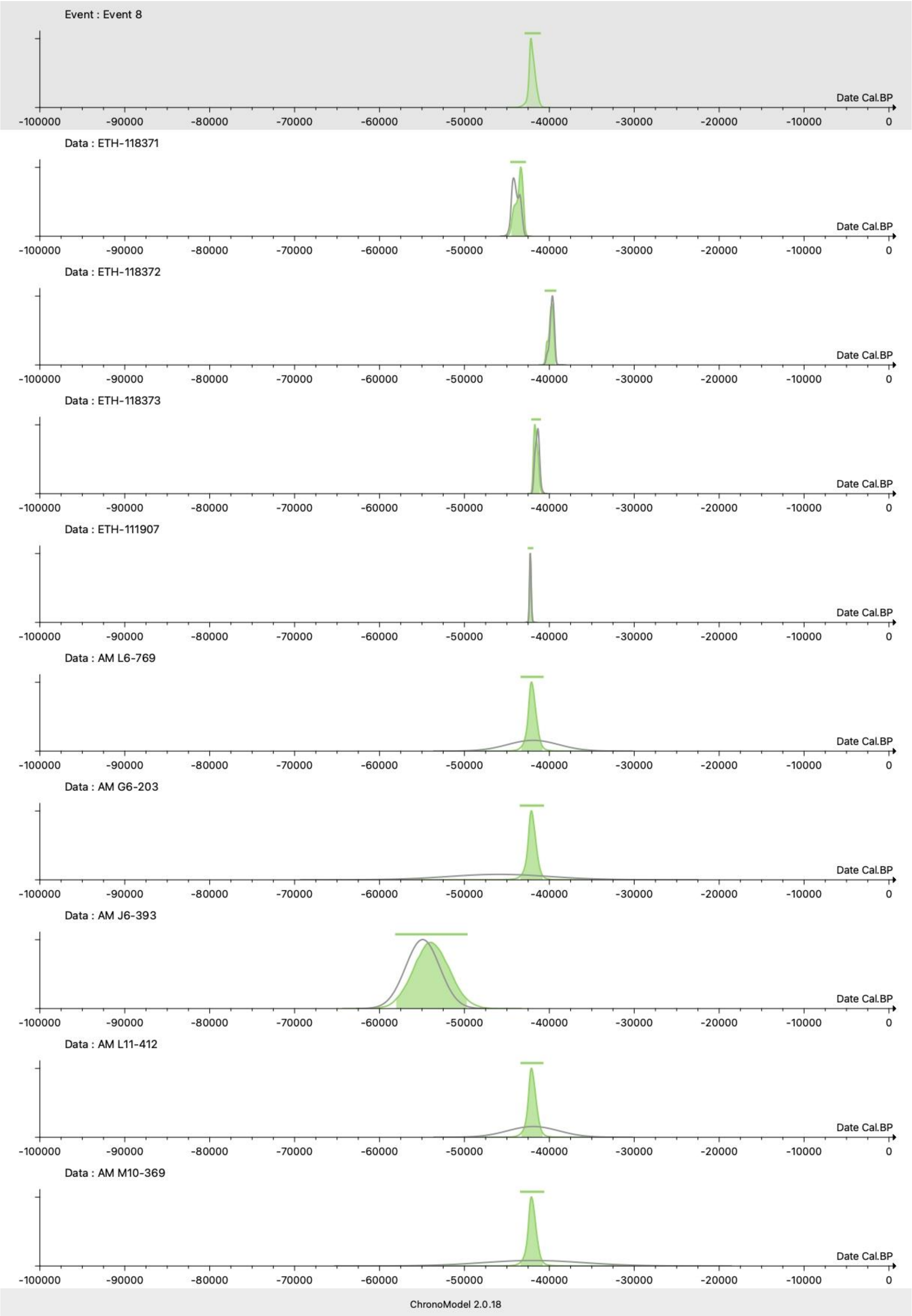

Fig. S3. Event 8.

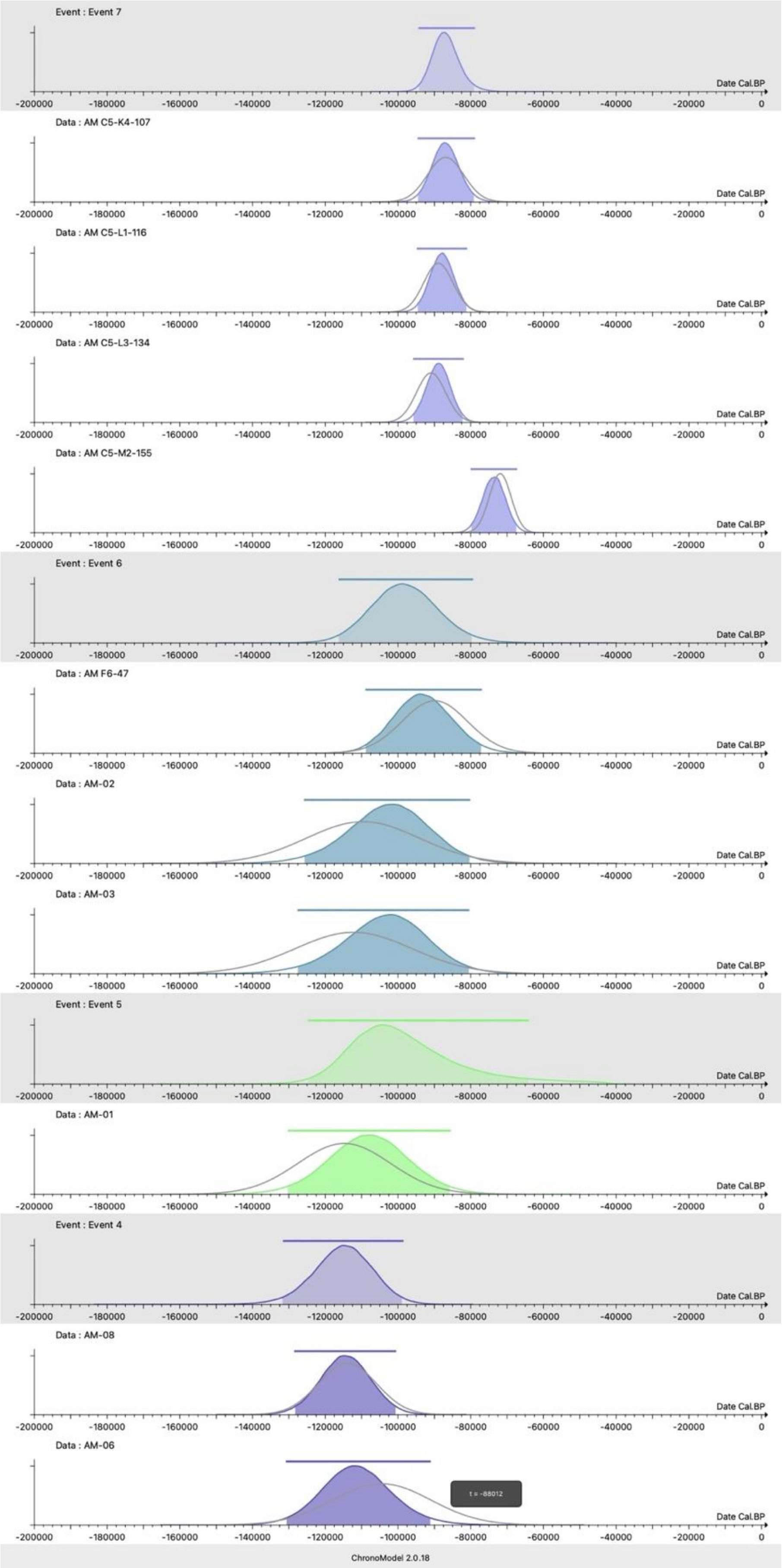

Fig. S4. Events 7, 6, 5 and 4.

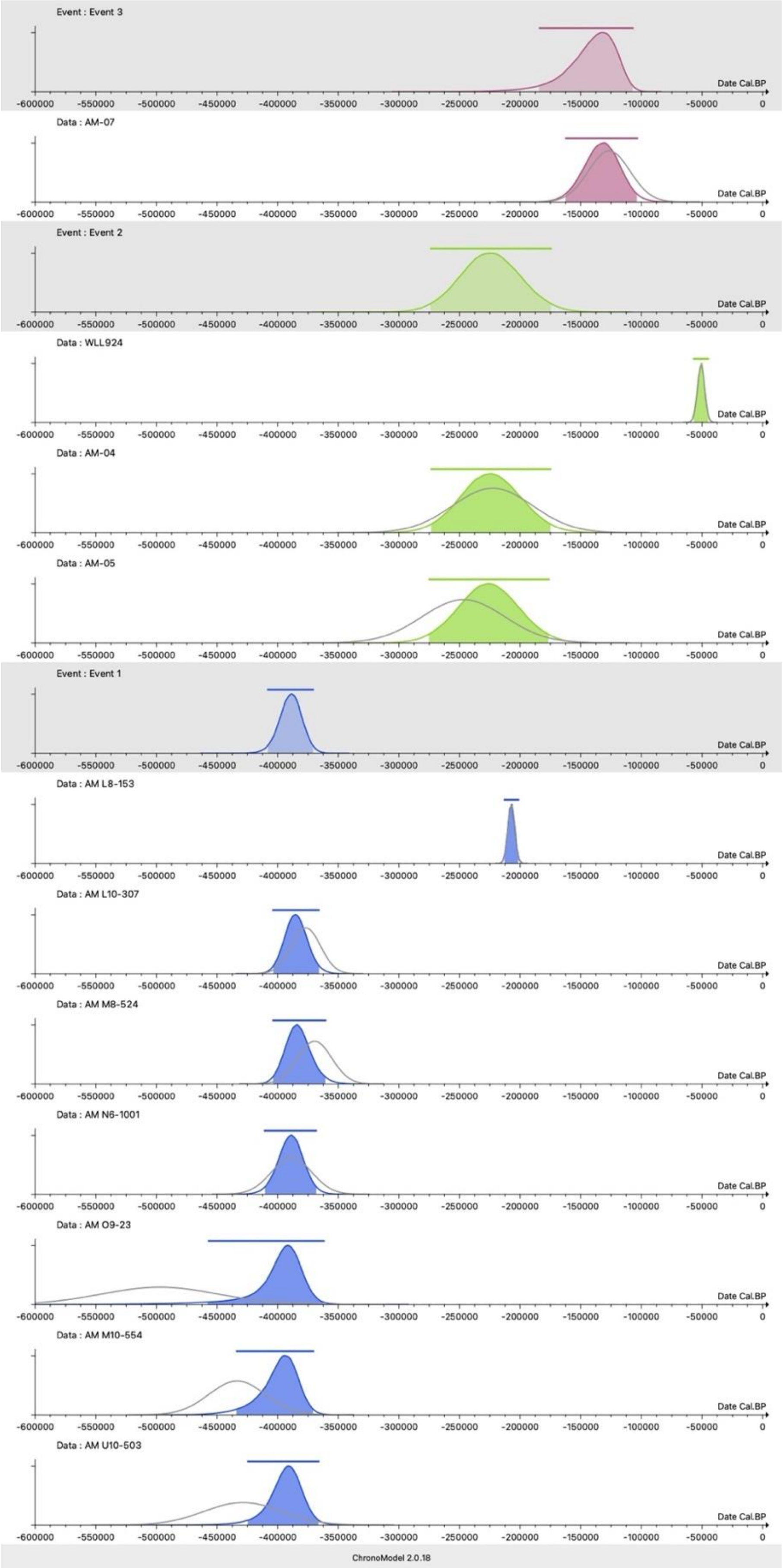

Fig. S5. Events 3, 2 and 1.
